# Hearing Loss and Preventable Hospitalizations

**DOI:** 10.1093/geroni/igab046.652

**Published:** 2021-12-17

**Authors:** Nicholas Reed, Emmanuel Garcia Morales

**Affiliations:** Johns Hopkins Bloomberg School of Public Health, Baltimore, Maryland, United States

## Abstract

Nearly half of all adults over the age of 60 years have hearing loss. Recent research suggests adults with hearing loss experience increased health care expenditures and hospitalization. However, little is known about whether these are preventable hospitalizations which may indicate poorer healthcare system engagement. In this cross-sectional analysis, we examined data from combined 2016-2018 Medicare Current Beneficiary Survey (MCBS) datasets. Participants are asked to describe their self-perceived trouble hearing. Preventable hospitalizations were defined and generated from administrative claims files based on the Agency for Healthcare Research and Quality identified conditions that should be manageable in ambulatory care settings. Multivariate regression models adjusted for demographic/socioeconomic characteristics and general health determinants were used to explore the association between trouble hearing and outcomes. The combined 2016-2018 MCBS administrative claims files included 18,814 participant-years, 49.8% reported no trouble hearing, 43.4% reported a little trouble and 6.8% a lot of trouble hearing, respectively. A higher proportion of those with a lot of trouble hearing (6.8%) experienced at least one preventable hospitalization compared to those with a little trouble hearing (3.4%) and no trouble hearing (2.5%). In a fully adjusted logistic regression model, hearing loss was associated with 1.35 times the odds of experiencing at least one preventable hospitalization per year (OR=1.35; 95% CI=1.03-1.77). Medicare beneficiaries with hearing loss experience higher rates of preventable hospitalizations. This may be due to avoidance of care due to communication barriers. Further work is needed to understand underlying reasons and whether addressing hearing loss modifies the observed association.

